# Dynamic changes in endoplasmic reticulum morphology and its contact with the plasma membrane in motor neurons in response to nerve injury

**DOI:** 10.1007/s00441-024-03858-x

**Published:** 2024-02-05

**Authors:** Mahmoud Elgendy, Hiromi Tamada, Takaya Taira, Yuma Iio, Akinobu Kawamura, Ayusa Kunogi, Yuka Mizutani, Hiroshi Kiyama

**Affiliations:** 1 Functional Anatomy and Neuroscience, Nagoya University Graduate School of Medicine, 65 Tsurumai, Showa-Ku, Nagoya, Aichi 466-8550 Japan; 2 Department of Anatomy and Embryology, Faculty of Veterinary Medicine, Damanhour University, Damanhour, 22511 Egypt; 3 Anatomy, Graduate School of Medicines, University of Fukui, Matsuokashimoaizuki, Eiheiji-Cho, Yoshida-gun, Fukui, 910-1193 Japan

**Keywords:** Endoplasmic reticulum, Endoplasmic reticulum-plasma membrane contact, Three-dimensional ultrastructure, Axon-injured motor neuron

## Abstract

The endoplasmic reticulum (ER) extends throughout a cell and plays a critical role in maintaining cellular homeostasis. Changes in ER shape could provide a clue to explore the mechanisms that underlie the fate determination of neurons after axon injury because the ER drastically changes its morphology under neuronal stress to maintain cellular homeostasis and recover from damage. Because of their tiny structures and richness in the soma, the detailed morphology of the ER and its dynamics have not been well analysed. In this study, the focused ion beam/scanning electron microscopy (FIB/SEM) analysis was performed to explore the ultra-structures of the ER in the somata of motor neuron with axon regenerative injury models. In normal motor neurons, ER in the somata is abundantly localised near the perinucleus and represents lamella-like structures. After injury, analysis of the ER volume and ER branching points indicated a collapse of the normal distribution and a transformation from lamella-like structures to mesh-like structures. Furthermore, accompanied by ER accumulation near the plasma membrane (PM), the contact between the ER and PM (ER-PM contacts) significantly increased after injury. The accumulation of extended-synaptotagmin 1 (E-Syt1), a tethering protein of the ER and PM that regulates Ca^2+^-dependent lipid transfer, was also identified by immunohistochemistry and quantitative Real-time PCR after injury. These morphological alterations of ER and the increase in ER-PM contacts may be crucial events that occur in motor neurons as a resilient response for the survival after axonal injury.

**Supplementary Information:**

The online version contains supplementary material available at 10.1007/s00441-024-03858-x.

## Introduction

The endoplasmic reticulum (ER) has continuous and elaborate networks distributed throughout each cell and performs a variety of crucial functions that help to maintain cellular homeostasis, such as Ca^2+^ storage, protein secretion, lipid synthesis and metabolite processing (Baumann and Walz 2001). The ER is a dynamic organelle that rearranges its structure to perform these functions (Friedman and Voeltz 2011). The ER is divided into ‘perinuclear ER’ or ‘periphery ER’ depending on their location (Westrate et al. 2015) and categorised into two patterns of shapes i.e. sheet structures and reticular structures, which are named as ER tubules in yeast (Voeltz et al. 2006; West et al. 2011). Especially in neurons, the stacks of the rough ER sheets known as ‘Nissl bodies (tigroids)’ are one of the characteristic structures that represent active protein synthesis, whilst the peripheral ER tubules are characterised by network formation, with branching points formed between the tubules (Schwarz and Blower 2016).

The identification of proteins and forces required to maintain ER shape has been reported, such as microtubules (Terasaki et al. 1986; Waterman-Storer and Salmon 1998), reticulon (Hu et al. 2008; McMahon and Gallop 2005; Voeltz et al. 2006) and atlastin (Bian et al. 2011; Hu et al. 2009; Orso et al. 2009). Several studies have explored the link between ER-shaping proteins and neurological diseases (Hetz and Saxena 2017; Saito and Imaizumi 2018; Sree et al. 2021). For instance, reticulon in Alzheimer’s disease (He et al. 2004) and atlastin in Hereditary spastic paraplegia (Wang et al. 2011) are not only the major proteins involved in ER shape regulation but also factors implicated in these diseases. Because the morphology of the ER can drastically change and is directly reflected by its functions, the details of these alterations may provide important clues for understanding physiological and pathological conditions (Westrate et al. 2015).

The ER network pervading the cytoplasm to reach almost every part within the cell has a close apposition contact with other organelles through membrane contact sites (MCSs), including mitochondria (Csordás et al. 2018; Csordás et al. 2006), lipid droplet (Jacquier et al. 2011) and plasma membrane (PM) (Phillips and Voeltz 2016). Amongst them, ER-PM contacts, where the ER is located in close proximity to the PM in a distance (≤ 30 nm) with neither membrane fusions nor vesicular transport structures, have been focused as a multiplicity of functions, including lipid transfer between the ER and PM (Gallo et al. 2016; Prinz et al. 2020; Saheki and De Camilli 2017a; Stefan 2020). Amongst the tethering proteins of the ER-PM, extended-synaptotagmins (E-Syts) are widely and commonly distributed in mammalian cells and yeast (homologue tricalbin) (Giordano et al. 2013; Min et al. 2007; Saheki and De Camilli 2017b; Schauder et al. 2014; Toulmay and Prinz 2012). E-Syts are different from synaptotagmins, which are the tethering proteins for vesicle secretion, in that it resides in the ER and tethers the ER to the PM via C2 domains without fusing each membrane (Chang et al. 2013). It has been reported that E-Syts regulate lipid transport with synaptotagmin-like mitochondrial lipid-binding (SMP) domains via Ca^2+^ concentration (Bian et al. 2018; Chang et al. 2013; Fernández-Busnadiego et al. 2015; Idevall-Hagren et al. 2015; Saheki et al. 2016; Yu et al. 2016).

MCSs, including ER-PM contacts, have been studied in the context of neurological diseases, such as amyotrophic lateral sclerosis (Nishimura et al. 2004), Huntington’s-like chorea (Landstrom et al. 2014), neuroacanthocytosis (Rampoldi et al. 2002) and Alzheimer’s disease (Area-Gomez et al. 2009). Although the alteration of ER morphology and ER-PM contact under neuronal damages have been studied, questions remain to be explored (Sree et al. 2021).

In this study, to explore the dynamics of ER morphology and ER-PM contacts responses in the somata of motor neurons in response to nerve injury, the 3D morphological analysis was performed using the hypoglossal nerve transection model as axonal regenerative injury model (Kiryu-Seo et al. 2022; Shishioh et al. 2022). Although there are some studies on ‘axonal ER’ after axotomy (Öztürk et al. 2020; Wu et al. 2017), the analysis of the ER in cell bodies has not been explored because organelle and membrane contacts in the soma are so rich that the resolution of light microscopy is unable to detect each structure. The analysis of a single plane using conventional transmission electron microscopy remains insufficient to determine their whole structures and measure them quantitatively (Scorrano et al. 2019). For this reason, the analysis using the focused ion beam/scanning electron microscopy (FIB/SEM) (Knott et al. 2008; Merchan-Perez et al. 2009; Narayan and Subramaniam 2015; Ohta et al. 2012; Tamada et al. 2020; Tamada et al. 2017; Tamada et al. 2021) was performed in the current study, because it can explore the 3D ultra-structures of the ER, sheet- or reticular-like structures, and their precise localisation in somata. In addition, FIB/SEM makes it possible to detect the ER-PM contacts as plates, which can lead to know about the different shapes and surface areas occupying in the PM (Wu et al. 2017). As we found alterations in the ER-PM contact after nerve injury by FIB/SEM in this study, we also addressed the alteration of E-Syts expression and localisation in response to nerve injury using immunohistochemistry and quantitative Real-time PCR.

## Material and methods

### Animals

All experiments were performed in accordance with the University Animal Care Guidelines for the Care and Use of Laboratory Animals and approved by the Nagoya University Institutional Animal Care and Use Committee. Ten- to twenty-two-week-old C57BL/6 mice of either sex (7 mice for FIB/SEM analysis,15 mice for immunohistochemistry and 14 mice for qRT-PCR analysis) were purchased from the SLC laboratory (Hamamatsu, Japan).

### Hypoglossal nerve injury

For hypoglossal nerve injury, the animals were anaesthetised with pentobarbital (45 mg/kg) via intraperitoneal injection. After dissecting a small skin incision and retracting the digastric muscles, the right hypoglossal nerve was exposed. The hypoglossal nerve was completely transected at its bifurcation using a pair of surgical scissors. The incision was sewn by nylon surgical sutures. Brain samples were collected 3, 7, 14 and 28 days after hypoglossal nerve transection.

### FIB/SEM specimen preparation

The mice were perfused using the half Karnovsky solution (0.05 M phosphate buffer containing 2% glutaraldehyde and 2% paraformaldehyde), and the brains containing the hypoglossal nucleus were removed. Trimmed specimens were immersed in the same prepared fixative solution for 2 h at 4 °C, followed by rinsing in the same buffer solution. Further specimens were fixed with 2% osmium tetroxide and 1.5% potassium ferrocyanide for 1 h at 4 °C. Subsequently, the specimens were washed with distilled water, treated with 1% thiocarbohydrazide, washed with distilled water, immersed in a solution of 2% osmium tetroxide for 1 h at room temperature, and again washed with distilled water. Next, for en bloc staining, the specimens were immersed in a solution of 4% uranyl acetate overnight at room temperature and washed with distilled water. Then, the specimens were furthermore stained with Walton’s lead aspartate solution at room temperature. Finally, the specimens were dehydrated in an ethyl alcohol series and ice-chilled acetone, embedded in epoxy resin (Epon812) then polymerised at 65 °C.

### FIB/SEM observation

After the resin block specimens were placed on a metal stub, the surfaces of the embedded specimens were exposed using a diamond-knife. Subsequently, the surfaces were coated with a protective layer of carbon in order to prevent charging artefacts, after that, the stub was fitted on the FIB/SEM stage (Scios., FEI Company, Hillsboro, OR., USA). After the deposition of carbon on the milling area, serial images of the block face were obtained through repeated cycles of sample surface milling and imaging using the Auto Slice and View G3 operating (FEI) software. Serial images of the block face were acquired by repeated cycles of sample surface milling using a focused gallium ion beam (FIB) at an accelerating voltage of 30 kV, current of 1.0 nA and milling step of 50 nm (for ER volume and branching analysis) or 20 nm (for ER-PM contact analysis). The image acquisition using SEM as a compositional contrast image from backscattered electrons at an accelerating voltage of 2.0 kV, current of 0.10 nA, dwell time of 3 μs and x pixel resolution of 19.27 nm/pix (for ER volume and branching analysis) or 5.62 nm/pix (for ER-PM contact analysis) was performed.

### 3D-structure reconstruction and volume analysis

Serial section images were reconstructed into three-dimensional (3D) images and analysed using the 3D visualisation software (Amira version 5.0–6.0.1, FEI Company).

### Statistical analysis

Statistical analysis was performed using a Sigma plot (12.0). Data were analysed using Student’s *t*-test and a two-way ANOVA analysis.

### Immunofluorescence staining

The mice were perfused with Zamboni’s fixative (0.1 M phosphate buffer containing 2% paraformaldehyde and 0.2% picric acid), and the brains were dissected. The trimmed samples were fixed in Zamboni’s fixative for 24 h at 4 °C. Then, the tissues were dehydrated using a 30% sucrose solution in 0.1 M phosphate buffer at 4 °C overnight, followed by embedding in the optimum cutting temperature (OCT) compound (Sakura Finetek, Torrance, Calif., USA) and frozen in dry ice. By using the cryostat microtome, the samples were cut into sections with the hypoglossal nucleus at a thickness of 14–16 µm and then mounted on adhesive glass slides. After antigen retrieval with HistoVT one (Nakarai tesque, Kyoto, Japan) for 10 min at 70 °C, the mounted sections were washed in 0.01 M phosphate-buffered saline (PBS), treated with 0.3% Triton X 100 in PBS for 20 min and blocked in 1% bovine serum albumin (BSA) for 20 min. Thereafter, slide mounted sections were incubated with the primary antibody goat anti-E-Syt1 (ab189199; Abcam, 1:100) and anti-Iba1 (#019–19741; Wako, 1:1000) at 4 °C overnight. Next, the sections were washed in 0.01 M PBS, followed by incubation with secondary antibodies conjugated with Alexa 488 (1:500; Donkey anti-Goat IgG, Invitrogen, Eugene, Oregon, USA) and Alexa 594 (1:500; Donkey anti-Rabbit IgG) for 3 h. Finally, the washed slides with 0.01 M PBS were mounted with FluorSave reagent (Merck Millipore). Images were obtained using a confocal laser-scanning microscope (Olympus FV10i; Tokyo, Japan).

### Quantitative real-time PCR (qRT-PCR)

The hypoglossal nuclei were individually collected from both the control and injured sides of 14 C57BL/6 mice 7 days after surgery. Total RNA was extracted using the acid guanidine isothiocyanate/phenol/chloroform extraction (AGPC) method. Total RNA was used to synthesise cDNA with Superscript III (Invitrogen, Carlsbad, CA). Subsequently, qRT-PCR was conducted on a StepOnePlus instrument (Applied Biosystems, Foster City, CA) utilising the Fast SYBR Green Master Mix (Applied Biosystems). The conditions for fast qRT-PCR reactions were as follows: 1 cycle of 95 °C for 20 s, 40 cycles of 95 °C and 60 °C for 30 s. Following PCR, a melting analysis was performed to verify the specificity of the amplicon. The results were then normalised to glyceraldehyde-3-phosphate dehydrogenase (GAPDH), and fold changes in gene expression were determined using the 2^(-ΔCt) method. Primer sets were as follows: *GAPDH* (sense 5′-TGACGTGCCGCCTGGAGAAA-3′, antisense 5′-AGTGTAGCCCAAGATGCCCTTCAG-3′), *Esyt1* (sense 5′-TGGGATCCTGGTATCTCAGC-3′, antisense 5′-CTGGGAGATCACGTCCATTT-3′), *Esyt2* (sense 5′-CGAATCACCGTTCCTCTTGT-3′, antisense 5′-GCTCTGGAAGATTTGGTTGC-3′), *Esyt3* (sense 5′-CAAGCCCTTCATAGGAGCTG-3′, antisense 5′-AGCAAATGGACTCGGATCAC-3′).

## Result

### Alteration of ER morphology in motor neurons after axon injury

**Fig. 1:**
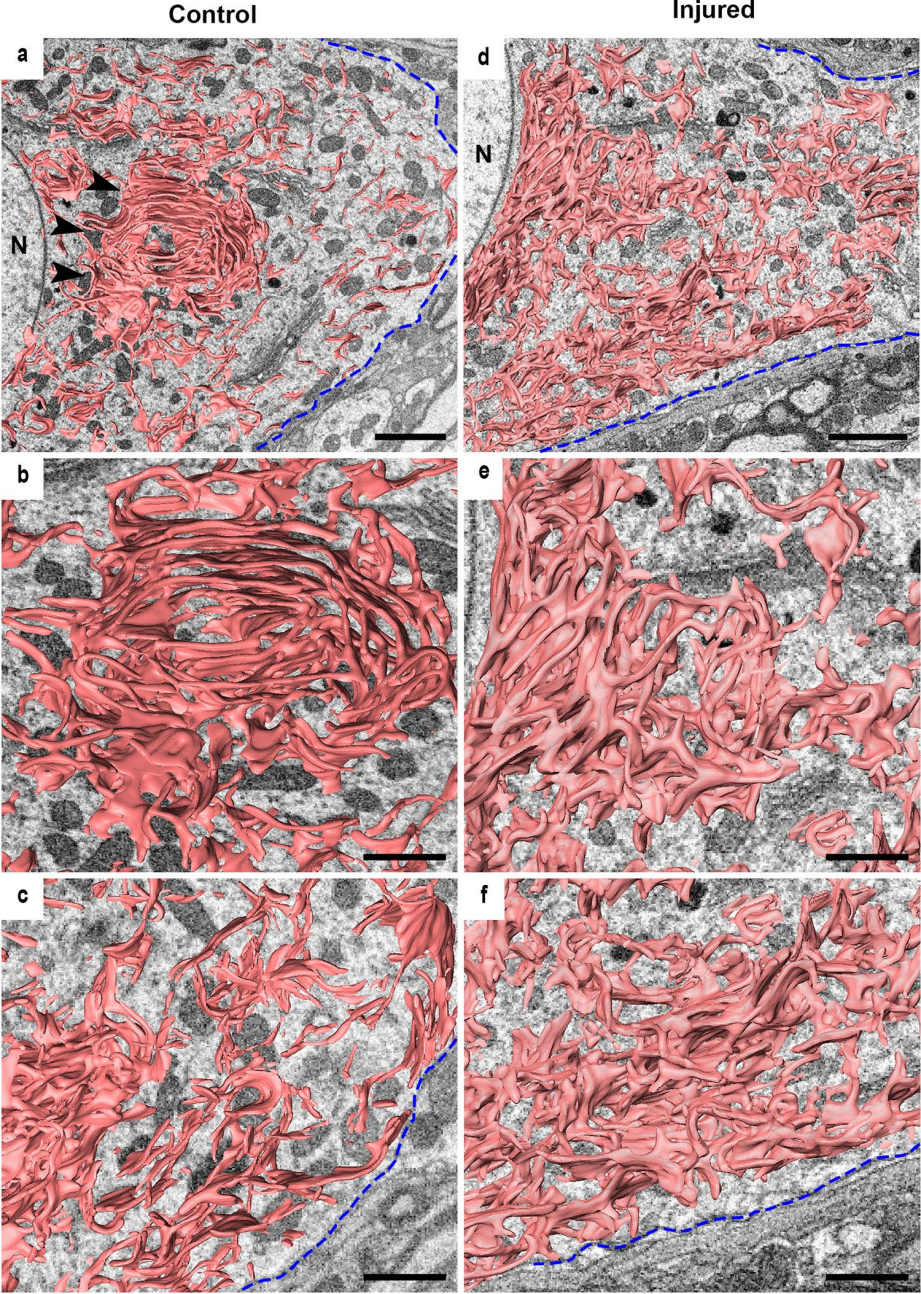
Three-dimensional images of ER after injury. **a** After reconstructing ER from serial images with FIB/SEM, the lamella-like structures (arrowheads) are detected around the nucleus (N) in the cell body. On the other hand, the distribution is sparse around the area close to the plasma membrane (PM) (blue dotted line). **b** Higher magnification three-dimensional images of ER around the nucleus in Fig. 1(a). **c** Higher magnification of Fig. 1(a) shows the three-dimensional images of ER close to the PM (blue dotted line). **d** In the injured cell at one week after injury, the ER-lamella structures are collapsed, and ER is distributed uniformly inside the neuron. The structure of the ER looks mesh-like structure throughout the cells (N: nucleus, blue dotted line: PM). **e** Higher magnification 3D images of the ER around the nucleus in Fig. 1(d). **f** Higher magnification 3D images of the ER form close to the PM (blue dotted line) in Fig. 1(d). Scale bar **a**, **d** 2 μm; **b**, **c**, **e**, **f** 1 μm

The characteristics of ER morphology in normal motor neurons of the hypoglossal nucleus include the existence of several lamellar or sheet-like ER stacks and uneven localisation in the cytoplasm with a tendency of a rich perinuclear region and less in the vicinity of the PM (Figs. 1a–c and S1; raw images before its segmentation). The FIB/SEM images show the typical lamellar or sheet-like ER stacks, which are comprised of extended regions of parallel and flat membrane bilayers stacked over each other (Friedman and Voeltz 2011). The structures were assumed to be Nissl bodies (tigroid substances), prominent units of the ER with adjacent ribosomes, and active sites for protein production in motor neurons. Furthermore, compared to the perinucleus area (Fig. 1b), the ER located around the peripheral regions (Fig. 1c), which is close to the PM, was sparse (Fig. 1a). When the axons were cut at the bifurcation site of the hypoglossal nerve, the characteristic lamella-like structures near the nucleus disappeared, and most of the ER structures became tubular or mesh-like, with many branching points (Fig. 1d–f). In addition, the mesh-like ER appeared to increase in the peripheral region near the PM (Fig. 1f); therefore, ER localisation in the cytoplasm appeared relatively even (Fig. 1d).

**Fig. 2:**
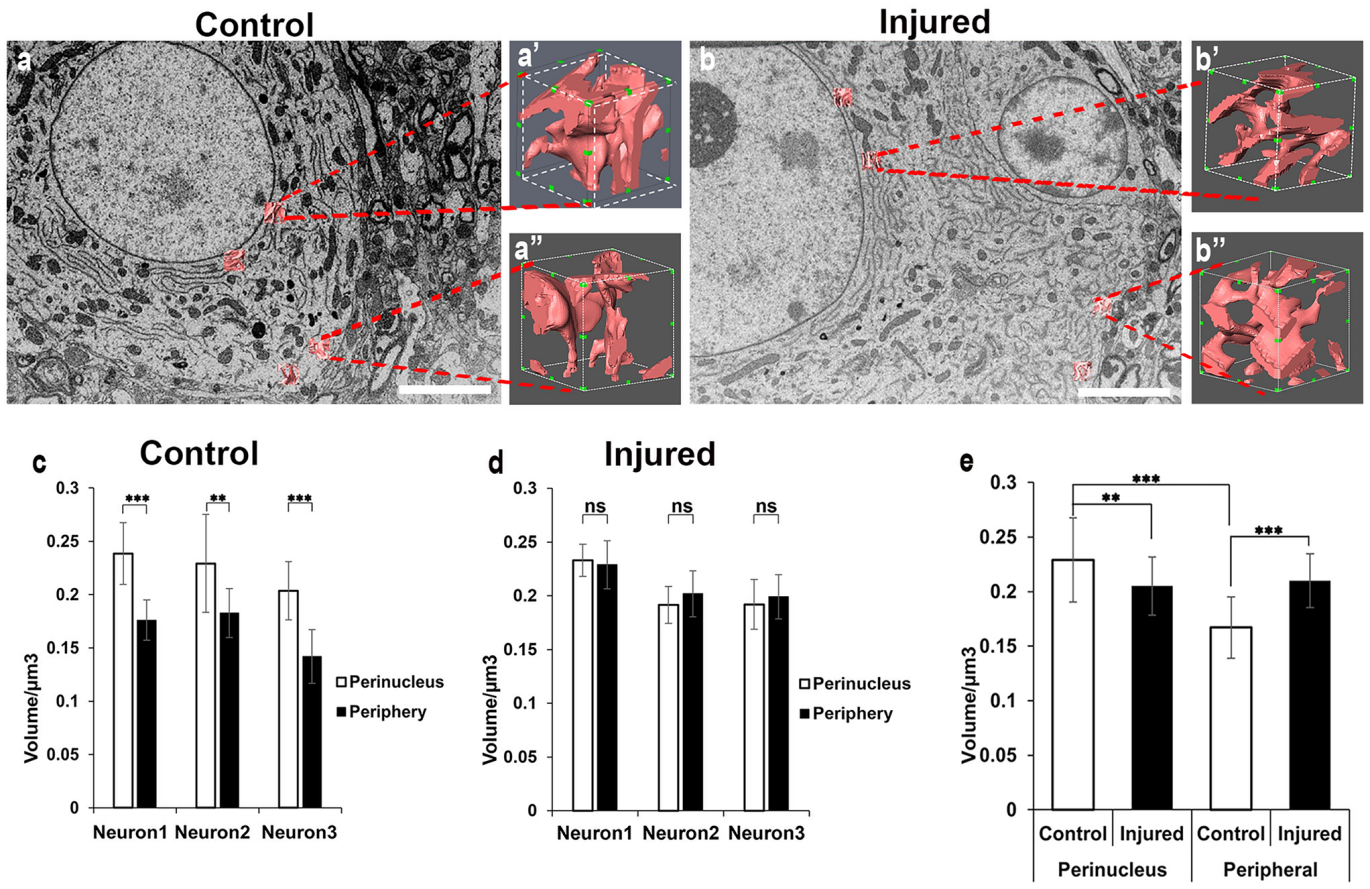
ER volume analysis in the control and injured motor neuron. **a**–**b** Representative three-dimensional reconstructed structures of the ER close to the nucleus (ER perinuclear) and close to the PM (ER periphery), which were selected randomly for volume analysis (**a** control neuron, **b** injured neuron at one week after injury). **a′**, **a″**, **b′**, **b″** Higher magnification of an example of a 3D reconstructed individual ER with a unit volume of 1 µm^3^. **c** The volume comparison of the ER perinuclear and ER periphery in the control motor neurons shows that the ER is more abundant in the area close to the nucleus than the area close to the PM. **d** In the injured motor neurons, the volume of ER perinuclear and ER periphery shows that there is no difference between them. **e** The comparison between the perinuclear ER region and the peripheral ER region in both control and injured motor neurons are conducted. Ten individual-ER from the perinuclear and peripheral regions in each cell were measured respectively (control: *n* = 3 neurons, *N* = 3 mice; injured: *n* = 3 neurons, *N* = 3 mice). The error bars show the standard deviation. The* p*-value **c**, **d** and **e** was determined using the Student’s *t*-test and two-way ANOVA with post hoc Tukey’s test respectively: **p < 0.005 and ***p < 0.001, (ns) = non-significant. Scale bar **a**, **b** 5 µm

To quantitatively analyse these morphological and distributional alterations, the comparison of ER volume between the perinucleus and peripheral regions was conducted, and the ER branching points were counted. First, the ER volume in a randomly selected unit volume (1 µm^3^) was measured (Fig. 2a′, b′ and a″, b″) for each ten regions of neuron at the perinucleus and periphery respectively (control: *n* = 3 neurons, *N* = 3 mice; injured: *n* = 3 neurons, *N* = 3 mice). In the control motor neuron (Fig. 2a), the ER volume around the nucleus was significantly larger (approximately 30%) than that at the peripheral regions (Fig. 2c; perinucleus vs periphery: control neuron 1, 0.239 ± 0.029 µm^3^ vs 0.176 ± 0.019 µm^3^; control neuron 2, 0.245 ± 0.046 µm^3^ vs 0.183 ± 0.023 µm^3^; control neuron 3, 0.204 ± 0.027 µm^3^ vs 0.142 ± 0.025 µm^3^; Student’s *t*-test *p* < 0.001, *p* < 0.005, *p* < 0.001 respectively). This indicated that the ER distribution was rich around the perinucleus regions in the control motor neurons and less in the distal or peri-PM regions. Conversely, in nerve-injured neurons (Fig. 2b), the ER dynamically changed their distribution and abundantly increased their volume in the area close appositional to the PM so that there was no significant difference between the ER perinucleus and ER periphery (Fig. 2d; perinucleus vs periphery; injured neuron 1: 0.233 ± 0.015 µm^3^ vs 0.229 ± 0.023 µm^3^; injured neuron 2: 0.192 ± 0.017 µm^3^ vs 0.202 ± 0.021 µm^3^; injured neuron 3: 0.192 ± 0.023 µm^3^ vs 0.199 ± 0.021 µm^3^; Student’s *t*-test *p*-values are non-significant). To confirm the changes in volume distribution at the perinuclear and peripheral ER levels after injury, a two-way ANOVA analysis was performed (Fig. 2e). This analysis revealed a significant increase in peripheral ER volume after injury, whereas perinuclear ER volume significantly decreased post-injury. As a result, the ER distribution within the injured motor neuron became more even (*p* < 0.001, two-way ANOVA with post hoc Turkey’s test).

**Fig. 3:**
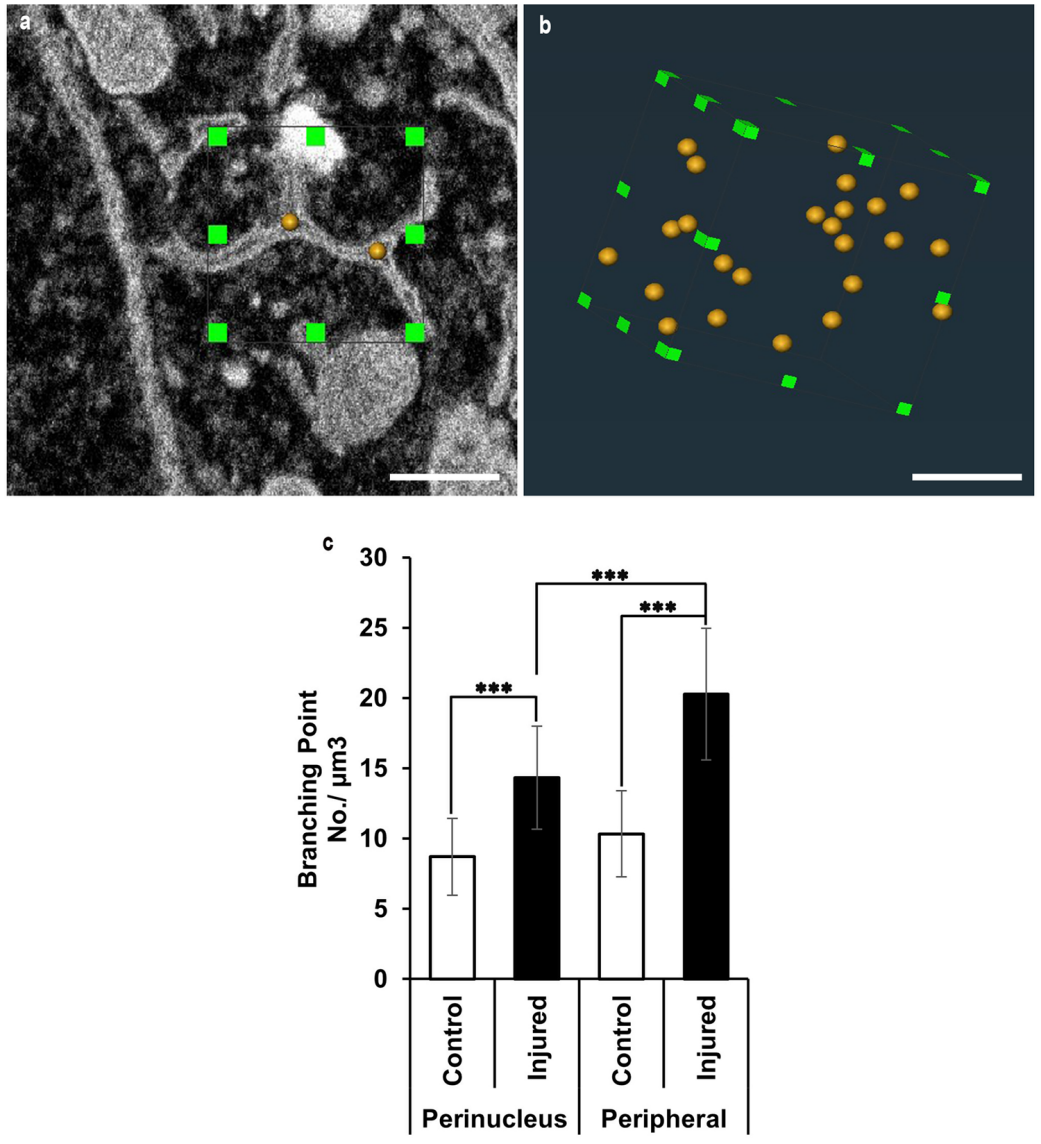
ER branching points analysis in the control and injured motor neuron. **a** Representative cross-sectional images of FIB/SEM. The yellow landmarks indicate the branching points. **b** The 3D unit volume (1 µm^3^) of the ER branching points analysis contains some branching points. **c** The branching points of the ER perinucleus and ER periphery are markedly increased in the injured motor neurons. Ten ER from each perinuclear and peripheral region in each cell (control: *n* = 3 neurons of three mice; injured: *n* = 3 neurons of three mice) were measured. The error bars show the standard deviation. The *p*-value was determined using the Two-way ANOVA with post hoc Tukey’s test: ***p < 0.001. Scale bar 500 nm

Next, the ER branching points were counted to quantitatively analyse the collapse of the lamellar structure and its transformation to mesh-like structures. The branching points in the ER within the unit volume (1 µm^3^) were plotted (Fig. 3a, b), and their numbers were compared between the control and injured neurons (Fig. 3c; for each control and injured analysis: ten ER perinuclear areas per neuron and ten ER periphery areas per neuron of three neurons from three mice). The analysis revealed that the average number of branching points in the control motor neurons was 8.7 ± 2.7 in the perinucleus regions and 10.3 ± 3.1 in the peripheral regions (Fig. 3c). In the injured motor neurons, these numbers significantly increased both in the perinucleus and in the periphery (14.3 ± 3.6 in the perinucleus, 20.3 ± 4.7 in the periphery in injured neurons; statistical analysis between the control and injury mice were performed with the two-way ANOVA with post hoc Turkey’s test; *p*-value < 0.001) (Fig. 3c). These results indicate that the transformation from lamella-like structures to tubular structures after an axonal injury is accompanied by branching, indicating that mesh-like structures become dominant throughout the somatic area.

### Increase of ER-PM contacts after injury

**Fig. 4:**
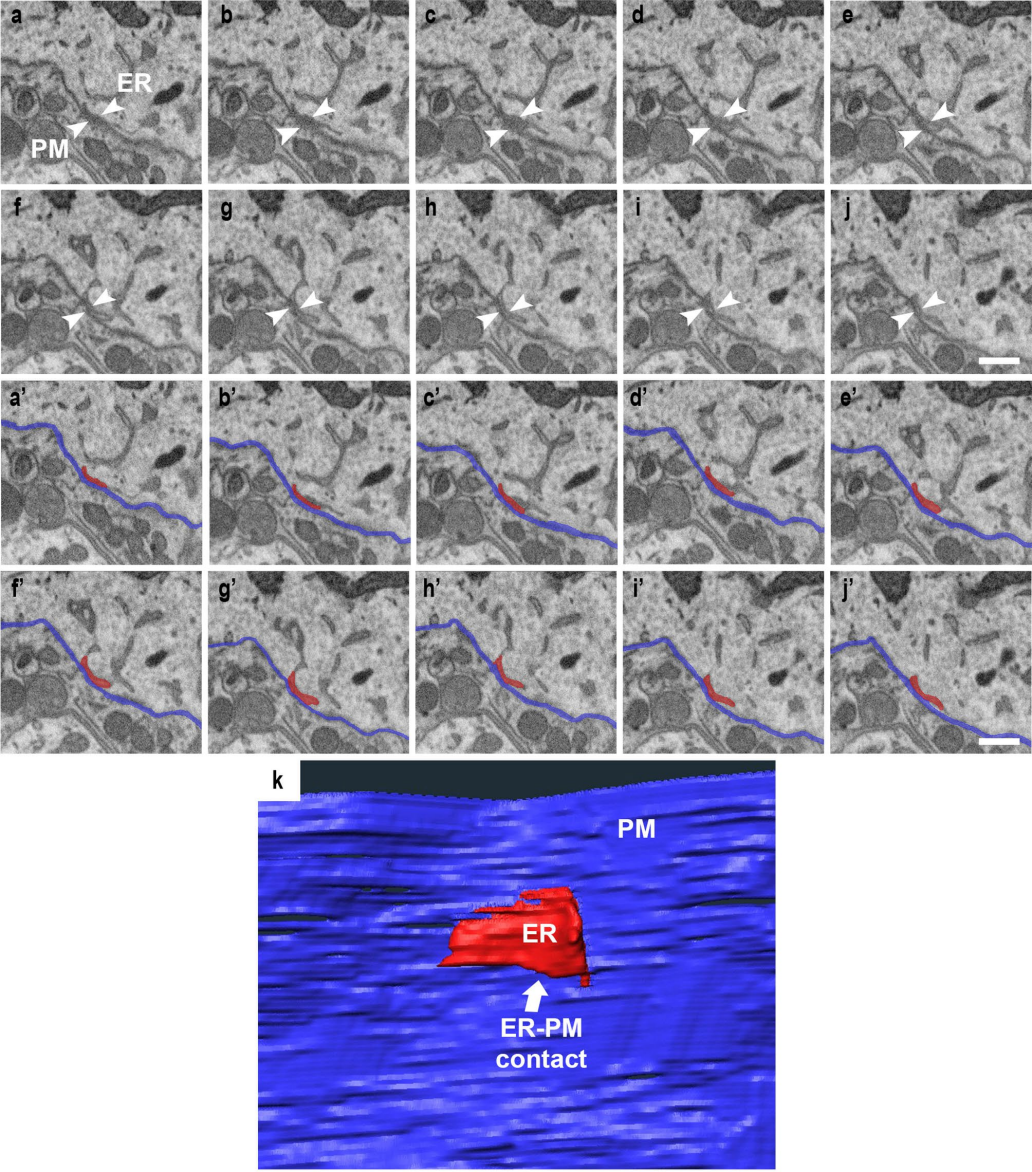
3D images of ER-PM contacts. **a**–**j** Representative serial images from FIB/SEM are showing the ER just beneath the PM. **a′**–**j′** The region of the ER (red) just attached along the PM (blue) is segmented for the reconstruction of the ER-PM contacts. **k** Showing a representative ER-PM contacts (arrows) is shown in 3D. Their shapes and sizes varied. Scale bar **a**–**c** 1 µm

**Fig. 5:**
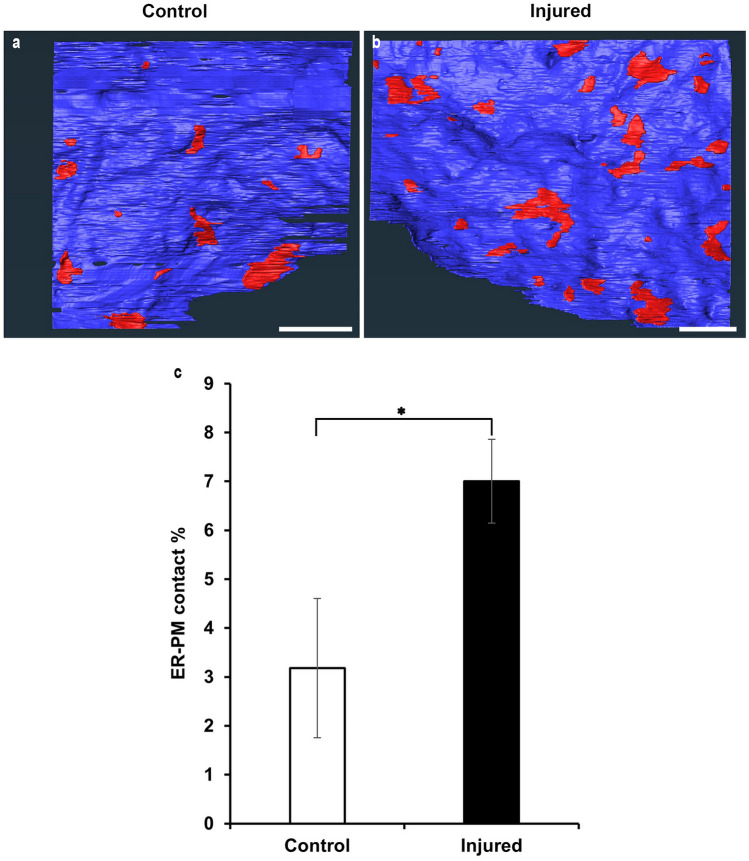
Increase in the ER-PM contacts after axon injury. **a** The ER-PM contacts in the cell body of control motor neuron were reconstructed. The blue sheet represents the PM, and red patch represents the reconstructed ER-PM contact. **b** The ER-PM contacts in the cell body of injured motor neuron were reconstructed. The blue sheet represents the PM, and each coloured patch represents the ER-PM contact. The number and area of the ER-PM contacts increased after axotomy. **c** The percentage of ER-PM contacts in the PM was calculated. The contact region significantly increased after injury, and the *p*-value was determined using the Student’s *t*-test: *p < 0.01 (*n* = 4 for control, *n* = 3 for injured). Scale bar 2 µm

Using the 3D ultrastructure obtained by FIB/SEM, the emergence of ER mesh-like structures and the acceleration of the peripheral distribution were explored. In addition to these two characteristics, we noticed that the tubular ER, which is located in the vicinity of the PM, became denser, and some of them appeared to attach to the PM. Next, we focused on whether ER-PM contacts varied in response to axon injury incident. The ER and PM contacts were observed in serial FIB/SEM images as two heterologous membranes in close apposition, which were never fused or invaded by other elements (Fig. 4a–j). With the segmentation of the ER just beneath and parallel to the PM(a′–j′), the ER-PM contacts could be reconstructed as plates of varied sizes and shapes (Fig. 4k). After reconstructing the PM and ER-PM contacts in randomly selected regions in both the control (Fig. 5a) and injured neurons (Fig. 5b), the surface areas were measured, and the percentage of ER-PM contacts per unit PM area was calculated. As a result, in the control cell bodies, 3.2 ± 1.4% of the PM was attached to the ER membrane (Fig. 5c; *n* = 4 neurons, *N* = 3 mice). On the other hand, in the axon-injured motor neurons, the percentage of contacts per PM was 7.0 ± 0.9% (Fig. 5c; *n* = 3 neurons, *N* = 3 mice). The results show that ER-PM contacts in the cell bodies were significantly increased after injury (Fig. 5c; Student’s *t*-test* p* < 0.01).

**Fig. 6:**
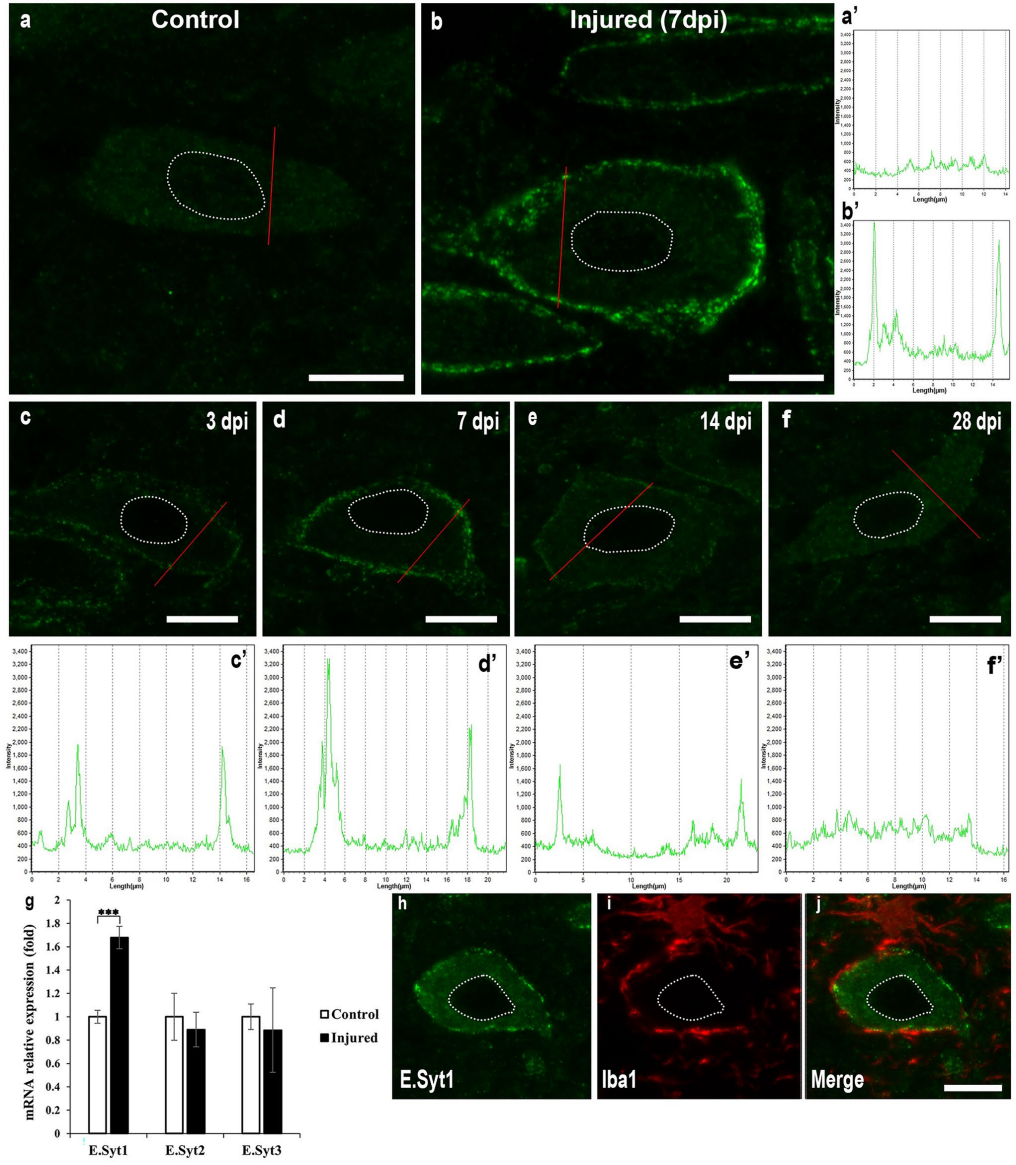
E-Syt1 accumulation observed along PM after axon injury. **a** Almost weak expression of E-Syt1 was detected in the control motor neurons. **b** E-Syt1 expression was detected along the PM of the injured motor neurons. **c**–**f** The transition in E-Syt1 expression after injury was observed from 3 to 28 dpi, the expression observed at 3 dpi until reach its peak at 7 dpi then little by little decreased. **a′**–**f′** Fluorescent intensities of E-Syt1 expression along the indicated lines were assessed in motor neurons before and after hypoglossal nerve transection. **g** qRT-PCR analysis was conducted on E-Syts mRNA extracted from both the control and injured sides of fourteen WT mice 7 days after surgery. The results have been normalised to the housekeeping gene GAPDH, and the values are presented as mean ± SD. The statistical analysis showed significant differences (***p < 0.0001) based on a two-way ANOVA with a post hoc Tukey’s test. **h**–**j** Representative image for the injured motor neuron (E-Syt1) surrounded by microglia (Iba1) which determined the boundaries of the motor neuron. Dotted red lines for the intensity fluorescent whilst dotted white circle determine the nucleus. *Scale bar* 10 µm

Next, we analysed E-Syt1 expression on ER-PM contacts in the somata of the hypoglossal nucleus using immunohistochemistry to support the morphological observations seen after injury. The injured motor neuron was identified by microglia, which wrapped their processes around the soma of the motor neuron (Fig. 6h–j). Although immunohistochemical analysis revealed low-density expression of E-Syt1 in the control neurons (Fig. 6a), strong expression was observed along the PM of injured motor neurons (Fig. 6b). The line intensity analysis indicates the significantly higher fluorescent intensity expression in the injured motor neuron restricted along the PM (Fig. 6b′) compared to the control motor neuron (Fig. 6a′). Furthermore, observations at several time points after injury revealed that the E-Syt1 expression appeared at 3 days post injury (3 dpi) to the peak at one week after injury, after that diminished gradually (Fig. 6c–f), which is also compatible with changes in the fluorescent intensity expression along the PM during the injury time course (Fig. 6c′–f′). To confirm the upregulation and accumulation of the Esyt1 post injury, we performed mRNA analysis by the *qRT-PCR* on Esyts mRNA extracted from both the contralateral and ipsilateral sides of wild type mice 7 days after hypoglossal nerve transection. The results clearly indicate a significant increase in E.syt1 mRNA expression in the injured motor neuron (*n* = 14, ***p < 0.0001, determined by a two-way ANOVA with a post hoc Tukey’s test).

## Discussion

The present study used FIB/SEM to allow 3D reconstruction of organelles, which further allows for statistical analyses of morphological changes in organelles. In this study, we demonstrated morphological alterations in the ER formation in motor neurons in response to axotomy. Our key findings were as follows: (1) ER sheet-like structures are transformed into mesh-like structures accompanied by a more homogeneous distribution after injury and (2) the increase of ER-PM contacts together with dominant localisation of the tethering protein E-Syt1 along the PM after injury.

### Alteration of ER morphology in response to axotomy

Our previous transcriptomic and proteomic analyses demonstrated that the expression of some subsets of proteins was downregulated in response to axotomy such as choline acetyltransferase (ChAT) whereas that of other subsets was upregulated as neuronal glutamate transporter EAAC1, DINE, ATF3, STAT3, c-Jun and so on (Kiryu et al. 1995; Kiryu-Seo et al. 2000, 2006; Kiryu-Seo and Kiyama 2011; Tanabe et al. 1999). Neuronal injury is assumed to stop normal neuronal metabolism and function, such as the synthesis and release of neurotransmitters, and induces the synthesis of some transporters and intracellular soluble proteins, which are necessary for stress responses (Moon 2018). Alteration of the molecular composition could be a critical response for neurons to survive after injury. To achieve this protein composition shift in response to axon injury, the change in ER formation from sheets to tubular-mesh and the simultaneous increase in free ribosomes within the cytoplasm might be important for changing the synthesis of protein species.

Some literatures have reported a close interaction between ER morphology shift and ER stress in several neuroinflammatory and neurodegenerative models (Hetz and Saxena 2017; Öztürk et al. 2020). Under cellular stress conditions, such as axon injury, ER homeostasis can be disrupted, and ER stress triggers an adaptive reaction known as the unfolded protein response (UPR) (Walter and Ron 2011; Wang and Kaufman 2016). In fact, in the process of axonal regeneration of the sciatic nerve, the expression of the ER stress-responsive chaperone BiP and the ER stress transducer XBP1s accelerates the axonal regeneration after injury (Oñate et al. 2016). Therefore, ER stress raised by axotomy could be one of the factors which promote the morphological shift of the ER.

Another advantage of the shift of ER formation from sheet to tubular mesh in response to axon injury would be the activation of inter-organelle lipid transfer (Vance 1990). Not only the ER-PM contacts observed in our study, but also mitochondria-ER attachments, the so-called mitochondria-associated ER membrane, appears to be altered in neurodegenerative diseases (Watanabe et al. 2016), suggesting that, together with lipid transfer, calcium metabolism could be regulated by changing the formation of ER from sheet to tubular mesh. Furthermore, it is possible that the ER walls or sheets in the cytoplasm are physically hazardous for molecular transport. For the diffusion of molecules in the cytoplasm, a tubular mesh-like structure rather than a sheet-like structure would be advantageous. This would easily allow the transfer of intracellular molecules from the PM to the nucleus or organelle to organelle.

### ER-PM contacts

Recently, the three-dimensional structures and spatial organisation of ER-PM contacts were examined using super-resolution live imaging techniques with ER-PM markers (Chang et al. 2013; Chen et al. 2019; Hsieh et al. 2017). To visualise the 3D architecture of ER-PM contact at molecular resolution, cryo-electron microscopy (cryo-ET) is used for optimally preserved vitrified cells (Fernández-Busnadiego et al. 2015). For the volume analysis of neuronal cells, FIB/SEM is advantageous for generating 3D reconstructions of ER-PM contacts by detecting membrane apposition (Wu et al. 2017).

According to the previous reports on 3D ER-PM analysis in the nucleus accumbens, the population of ER-PM contacts per total PM area was 12.48% (Wu et al. 2017) in normal neuronal somata, although our results from motor neurons were lower than that percentage. This difference is assumed to be derived from neuronal types (Sree et al. 2021). Intriguingly, the ER-PM contact areas in the PM of motor neurons became almost double in response to axon injury, which is similar to the previous studies that reported an increase in ER-PM contacts in response to disturbances in calcium influx (Chang et al. 2013; Poteser et al. 2016; Wu et al. 2006).

Thus far, ER-PM contacts have been suggested to do multiple functions (Dong et al. 2016; Gallo et al. 2016; Stefan et al. 2013). In order to construct ER-PM contacts, one of the critical tethering proteins is E-Syt1, and the present study clearly demonstrated a shift in E-Syt1 localisation to the PM in response to axotomy. E-Syt1 possesses two functional domains, the C2 and SMP domain; the C2 domain interacts with PM phosphoinositide PI (4,5) P_2_ depending on the elevation of cytosolic Ca^2+^, and the SMP domain is associated with the exchange of lipids between the ER and PM (Giordano et al. 2013; Jeyasimman and Saheki 2020; Reinisch and De Camilli 2016; Schauder et al. 2014; Toulmay and Prinz 2012). In addition to these two functional domains, E-Syt1 also plays a role in lipid transfer and Ca^2+^ metabolism. Efficient removal of diacylglycerol (DAG) from the PM to the ER is regulated by E-Syts (Saheki et al. 2016). Since axotomy disturbs Ca^2+^ homeostasis (Gemes et al. 2009; Li et al. 2013), E-Syts regulation of the ER-PM contact might mediate PM organisation, leading to repair after injury in our model.

Recently, it was reported that in yeast, tricalbins (Tcbs), orthologues of mammalian E-Syts, regulate plasma membrane phospholipid homeostasis in response to PM integrity stress, such as heat stress condition (Collado et al. 2019). Although Tcbs is not the main component for maintaining phospholipid homeostasis under normal growth conditions, under the stress condition with increasing cytoplasmic Ca^2+^ levels, it is critical for transporting lipids from the ER to the PM (Omnus et al. 2016; Thomas et al. 2022). In terms of axon injury stress, many literatures demonstrated the changes in the expression of molecules associated with lipid metabolism (Roy and Tedeschi 2021; Shishioh et al. 2022). It is likely that locally synthesised phospholipids on one organelle membrane are transferred to other organelle membranes via direct membrane contact to maintain the integrity of neurons against injury.

The ER-PM junction is also known as a critical site where the store operated Ca^2+^ entry (SOCE) occurs (Trebak and Putney 2017). Following the release of Ca^2+^ from the ER, SOCE replenishes the depleted intracellular Ca^2+^ stores (Schulte and Blum 2022). An increasing number of recent findings have demonstrated that these ER-PM junctions serve as centres for the assembly of Ca^2+^ signalling complexes (Courjaret et al. 2023). Axon injury induces calcium release from intracellular storage, such as the ER, which leads to an increase in the cytosolic calcium concentration, starting the early signal for the soma response; therefore, low Ca^2+^ stock levels in the ER may continue for some period (Khaitin 2021; Rishal and Fainzilber 2014). To avoid calcium imbalance in neuron, compensation of the low Ca^2+^ stock level in the ER through the activation of SOCE would be necessary by expanding the ER-PM junction. This might be another reason why the ER-PM area needs to increase in motor neurons after axotomy.

### Interaction between ER morphology and ER-PM contacts

Alterations in ER morphology and ER-PM contacts were detected simultaneously in this study, suggesting the interactions and regulatory mechanisms with each other. In yeast, it has been known that the peripheral ER tethering to the PM (called ‘cortical ER’) is mainly a tubule-like structure rather than a sheet-like structure (Baumann and Walz 2001; West et al. 2011). It has also been reported that the actin regulator of ER structure (actin-binding protein Filament A: FLNA) and the regulator of ER (protein kinase RNA-like ER kinase: PERK) interact closely and regulate ER-PM contact via tethering with E-Syt1 (van Vliet et al. 2017). Other literatures reported that the depletion of the ER-PM contact secondarily triggers ER morphological alterations and UPR stress (Manford et al. 2012).

The interaction between ER morphology and ER-PM contact has also been studied under neuronal stress. Using Caenorhabditis elegans cholinergic DA9 motor neurons, in the mutant ER-shaping proteins CIL-1 and ATLN1, not only abnormal balance of ER sheet and tubules but also abnormal localisation of E-Syt2 distribution are observed (Sun et al. 2021). Furthermore, under laser-axotomy condition, axon regeneration was suppressed in the mutants. Based on these results, authors suggest that these ER-shaping proteins maintain neuronal resilience against damage through ER-PM contact. Referring to these studies, the appearance of an ER mesh-like structure, that is, a tubular ER, and the accumulation of ER-PM contacts after injury in our model might occur concurrently and coincidentally.

## Conclusion

Our study with FIB/SEM directly showed the dynamic alterations in the ER morphology and an increase of ER-PM contact, which is perhaps E-Syt1-dependent, in the motor neuron after axotomy. The tubular mesh structure of the ER throughout the cytoplasm may be beneficial for motor neurons to function in a resilient response. As for the increase of ER-PM contact, our observations suggest that the replenishment of depleted intracellular Ca^2+^ stores at ER-PM contacts in the somata might be required for survival or regeneration after injury. At present, it is unclear whether ER-PM mediated lipid transfer is necessary for survival or axon regeneration processes, and what lipid species are transported via ER-PM contact. Although some questions remain regarding the alteration of ER morphology, these changes might be critical for maintaining the integrity of motor neurons and promoting regeneration processes.

### Supplementary Information

Below is the link to the electronic supplementary material.

Supplementary file1 (TIF 2978 KB)

## Data Availability

All relevant data are available from the corresponding authors.
